# Standardized Mortality Ratios (SMRs) and Radon Exposure Analysis for Lung Cancer and All-Cause Mortality in Locorotondo, Southern Italy

**DOI:** 10.3390/medicina61010047

**Published:** 2024-12-31

**Authors:** Giovanni Maria Ferri, Luigi De Maria, Giuseppe Delvecchio, Antonio Caputi, Stefano Sole, Gianmarco Giannelli, Gianfranco Sifanno, Ilaria Maria Di Somma, Floriana Pentimone, Domenica Cavone, Angela Stufano, Piero Lovreglio, Vitantonio Ricci, Luigi Vimercati

**Affiliations:** 1 Interdisciplinary Department of Medicine, University of Bari, Piazza G. Cesare, 11, 70124 Bari, Italy; giovannimferri@gmail.com (G.M.F.); luigi.demaria@uniba.it (L.D.M.); giuseppe.delvecchio1@uniba.it (G.D.); antonio.caputi@uniba.it (A.C.); stefano.sole@uniba.it (S.S.); gianmarco.giannelli@uniba.it (G.G.); gianfranco.sifanno@uniba.it (G.S.); ilaria.disomma@uniba.it (I.M.D.S.); floriana.pentimone@uniba.it (F.P.); domenica.cavone@uniba.it (D.C.); angela.stufano@uniba.it (A.S.); piero.lovreglio@uniba.it (P.L.); 2 Prevention Department, Local Health Authority Bari, Viale Bari, 28, 70011 Alberobello, Italy; vitantonio.ricci@asl.bari.it

**Keywords:** radon exposure, lung cancer mortality, all-cause mortality, epidemiology, Southern Italy

## Abstract

*Background and Objectives*: Radon is a known risk factor for lung cancer, and residential radon exposure is the leading cause of lung cancer in never smokers; however, in Italy, there is still a lack of public awareness regarding the risk caused by residential radon exposure. In this mortality study, which was carried out in an Italian Apulian town (Locorotondo) of the Bari province, we aimed to analyze lung cancer mortality and all-cause mortality in a population highly exposed to radon. *Materials and Methods*: The study period was 1998–2021. Local and Italian population and national mortality data were collected from the Italian National Institute of Statistics (ISTAT) website platform. Local mortality data were collected using copies of the Local Health Authority death certificates. *Results*: We identified 117 lung cancers in the studied period. The mortality data trends revealed a decrease in the all-causes standardized mortality ratios (SMRs), increases in the incidence rates of lung cancer and colorectal cancer in recent years, and a decrease in the incidence of noncancer diseases. We also found high SMRs for colorectal cancer until 2016 among older females. With respect to the cardio-circulatory system, only in 2014 did the male SMRs significantly influence the total SMR; after this period, a decreasing stable trend was observed. *Conclusions*: The natural balance of the population is decreasing, and mortality is decreasing for all causes. A future study will be needed to assess the associations between observed lung cancer cases and domestic radon exposure to drive radon mitigation and public health strategies.

## 1. Introduction

Mortality rates from all cancers and the most common cancer sites have declined over the last 25 years in many countries, except for cancers of the pancreas and lung (in women) [1]. According to the International Agency for Research on Cancer (IARC), in Italy in 2022, the three most common cancers in both sexes were breast (13.2%), colorectal (12.6%), and lung (10.0%) cancers [2]. In addition to other risk factors (e.g., environmental and occupational exposures, chronic lung disease, lung infections, and lifestyle factors), the main risk factor for lung cancer is tobacco smoking. Therefore, the primary prevention of this disease focuses on fighting smoking [3].

With respect to the risk evaluation of colorectal cancer (CRC) risk, there is support for a large amount of scientific production. For example, some genetic factors linked to the different locations of intestinal cancer between the two sexes have been highlighted (right sections in women and left sections in men). The genetic factors for right-sided (proximal) cancer are based on microsatellite instability (MSI). This phenomenon is produced by the CpG island methylator phenotype (CIMP), chromosome 7 gene BRAF mutation, MAPK signaling, and the serrated mutagenic pathway of the mutagenic CYP450 metabolite HNPCC [4]. Estrogen exposure is a protective factor against MSI, and a lack of estrogen in older women increases the risk of colon cancer. Hormone replacement therapy (HRT) is able to reduce this cancer risk [5]. The increased risk due to high CIMP is also evident for caecum cancer [6]. The female sex is positively associated with the methylation of a tumor suppressor gene (p161nk4a) [7]. Vascular endothelial growth polymorphisms increase the risk of colon cancer in women [8]. Right-sided colon cancer, which is associated with a poor prognosis, is more common in women [4]. Furthermore, the greater length of the colon is also linked to high fiber consumption [9], and some types of right-sided colorectal neoplasms (flat and depressed types) [10] are more common in women, reducing the sensitivity and specificity of screening tests and/or endoscopic examinations and increasing the risk of cancer. Moreover, just as a high-fat diet increases the incidence of right-sided colon cancer, high protein consumption is associated with left-sided colon cancer [11,12], and carbohydrate consumption is associated with colon cancer in women and with rectal cancer in men [13]. The intake of fat, cholesterol, sucrose, and lactose is associated with right-sided colon cancer [14]. In contrast, intake of iron, calcium, and 25-hydroxyvitamin D are inversely associated with distal colon cancer [15,16,17,18]. Among occupational risk factors, working shift work with night shifts for ≥10 years is associated with a higher risk of colorectal cancer (HR = 1.64, 95% CI95% 1.01–2.66) [19].

This study follows the results of two previous studies conducted in the same geographical area: the first was carried out in several towns of the two Apulian provinces of Bari and Lecce, related to the correlation between radon and lung cancer [20], and the second involved the validation of new sensors for radon detection and the association between radon exposure and earthquakes [21]. These studies revealed that the sites in the province of Bari with higher estimated risks of lung cancer associated with radon were Gravina di Puglia and Locorotondo, with estimated incidence rates of lung cancer of 5.36 and 3.44 per 10,000 inhabitants, respectively. These estimates need to be verified through mortality studies to assess the association between radon exposure and lung cancer. Locorotondo is an appropriate setting for this purpose because it has been identified as one of the Apulian municipalities most subject to the phenomenon of “land consumption” due to concreting. Among the rural areas, this small municipality has the highest number of domestic volumes compared with the number of inhabitants. Furthermore, most houses have basements with high exposure to radon decay products [20,21].

In this mortality study, which was carried out in Locorotondo, we aimed to analyze lung cancer mortality and all-cause mortality in a population highly exposed to radon.

## 2. Materials and Methods

This mortality study was carried out in an Italian Apulian town (Locorotondo) of the Bari province. Local and Italian population data and national mortality data were collected from the Italian National Institute of Statistics (ISTAT) website platform (http://www.istat.it/en/news/mortality-data/, accessed on 20 August 2024) and from the Local Health Authority (ASL) death certificate copies. The period of the study was 1998–2021. The authorization of the Director of ASL of the province of Bari and the responsible physician of the Public Health Office of Locorotondo was requested and obtained.

All the data related to all the causes of death (initial and others), sex, residence, and other information were reported in the database. The data analysis was based on the computation of the crude death rates by the six most important causes of death selected after the analysis of the observed highest simple frequencies: all causes, malignant cancer, lung cancer, colorectal cancer, all non-malignant cancer causes, and cardiovascular diseases. The standardized mortality ratio (SMR) is the ratio of the observed number of deaths to the expected number of deaths in the study population under the assumption that the mortality rates for the study population are the same as those for the general population. The indirect age adjustments of the SMRs were performed using three different methods. The first measurement of the SMRs was performed using the method table based on Haenszel, W. [22] and explained by Breslow and Day [23]. The user is prompted to enter observed and expected numbers of deaths in the respective data entry cells. The computed confidence limits were 95%. The lower and upper limits for the observed deaths were extracted from the tabulated values (Table 1) [24].

For example, in Locorotondo, the SMR for lung cancer in males in 2009 was as follows:

SMR = Observed cases (9)/Expected cases (6.3) = 1.43

The confidence limits were as follows:

OL = Observed cases (9) × Table lower limit factor (0.46) = 4.12

Ou = Observed cases (9) × Table Upper Limit Factor (1.90) = 17.10

95% LCL (lower SMR 95% confidence limit) = OL (4.12)/Expected cases (6.3) = 0.65

95% UCL (upper SMR 95% confidence limit) = Ou (17.10)/Expected cases (6.3) = 2.71

SMR = 1.43 (0.65–2.71)

These estimates were computed using a personally created calculator based on the methods of Haenszel and Breslow [22,23].

The second (A) and third (B) SMR measurement methods were based on the choice to use 90% confidence limit computations because, in many cases, the number of observed deaths was very low; therefore, the use of the standard 95% was inadequate. We used the software “OPENEPI 3.01” based on several different methods. Please note that the observed number of cases must be an integer, as they are assumed to be Poisson variates (random variables with a Poisson distribution). The user can change the confidence interval settings as seen in the data entry dialog box (or) in ‘Options/Setting’ on the main menu screen. The output from the example above is as follows: two *p* values are calculated under the assumption that the observed deaths are Poisson variates (random variables with a Poisson distribution) and that the expected deaths are unchanged. Exact confidence intervals and *p* value should be used when the number of observed deaths is less than or equal to five. For greater numbers of observed deaths, approximation methods are nearly as accurate as exact tests are. In the output window, the statistical significance test between the observed and expected number of deaths based on the Mid-P exact method yielded *p* = 0.6571. The point estimate of SMR is 1.212, and six different methods are used to calculate the confidence interval around this estimate: the Mid-P exact test, Fisher’s exact test, the normal approximation, the Byar approximation, the Rothman/Greenland method, the Vandenbroucke method, and the 3 Ury and Wiggins method. Among these methods, the Mid-P exact test is generally the preferred method. Based on *p* values and confidence intervals that include a null value of ‘1’ in the output table, the interpretation is that there is no significant excess or deficit in the mortality rate in the study population compared with that in the general population. For confidence limit estimates of <0.0, the value 0.0 is shown. All confidence intervals calculated are two-sided and depend on the setting of the user’s choice (90%, 95%, 99%, 99.9%, or 99.99%). The formulas for the methods are as follows:

- Mid-P exact test (see Rothman and Boice)
  Lower bound: $\frac{1}{2}\left(\frac{e^{-\bar{a}}{\bar{a}}^{a}}{a!}+\overset{a-1}{\underset{k=0}{\sum }}\frac{e^{-\bar{a}}{\bar{a}}^{k}}{k!}\right)=1-\frac{\alpha }{2}$
  Upper bound: $\frac{1}{2}\left(\frac{e^{-\bar{a}}{\bar{a}}^{a}}{a!}+\overset{a-1}{\underset{k=0}{\sum }}\frac{e^{-\bar{a}}{\bar{a}}^{k}}{k!}\right)=\frac{\alpha }{2}$
- Fisher’s exact test (see Rothman and Boice)
  Lower bound: $\sum {\left(k=0\right)}^{a}\left(e^{\left(-ā\right)}*{ā}^{k}\right)/k!=1-\alpha /2$
  Upper bound: $\sum {\left(k=0\right)}^{a}\left(e^{\left(-ā\right)}*{ā}^{k}\right)/k!=\alpha /2$

Based on the Monte Carlo simulation results, the LMP test (Lancaster’s Mid-P test) outperformed the FET (Fisher’s exact test) across a wide range of conditions typical of adverse impact analyses. The LMP test was also found to provide better control over Type I errors than the large-sample Z test when the sample size was very small, but it tended to have slightly lower power than the Z test under some conditions (Biddle D.A., Morris S. B. 2011). Analytical methodological descriptions have been previously published [25,26]. The explanation of the SMR’s significance was based on the principle of “falsification of Popper” but not on the idea “against the probability inductive statistical approach”. Our use of the probability approach is the most widespread interpretative tool among statistical science workers and is based on the probability test for the falsification of the null hypothesis (H0).

## 3. Results

### 3.1. Characteristics of the Sample

The analysis of data related to the Locorotondo population over the period of 1998–2021 revealed a series of changes. In particular, there was a slight increase in the population of 139 people (from 13,927 to 14,066). The number of people aged 0–4 years decreased by −0.6% (from 609 to 535), with a percentage reduction of −0.7% in males (from 54.0% to 53.3%) and a percentage increase of +0.7% in females (from 46.0% to 46.7%). In the population aged 95 or over, there was an increase of +0.2% (from 16 to 37), with a percentage decrease of −13.2% in males (from 37.5% to 24.3%) and a percentage increase of +13.2% in females (from 62.5% to 75.7%). The study population included 5676 single subjects, 7054 married, 986 widowed, and 350 divorced. In 2021, there was a substantial increase in the number of people aged 45–49 years to ≥95 years and a decrease in the number of people aged 0–4 years to 40–44 years. The population was older in 2021 than in 2002 (Table 2). The analysis of the natural growth rate (the ratio between newborns and the average population of that year per thousand individuals) revealed a negative trend, with a large negative increase in the difference between births and deaths in the Locorotondo population from 2002 to 2022. In 2022, there were 164 deaths out of 104 births (−60 persons). All the data are described in Table 2. Finally, the overall stability of the population in Locorotondo (2017–2022) is strong because the annual mean variability is very low (−0.36%). In 2022, the total balance (natural balance–migration balance) was −41 (UGEO source: https://ugeo.urbistat.com/AdminStat/it/it/demografia/dati-sintesi/locorotondo/72025/4?Export=2&Export=1&Export=2&MasterType=1, accessed on 20 August 2024).

### 3.2. Mortality Data [SMRs]

For the standardized mortality ratio (SMR) computations, all the described methods were used. We decided to use the Mid-P exact test (90% CI) for the description of the results because many of the observed numbers of deaths were very small, and this method was stronger in this case.

#### 3.2.1. All-Cause Mortality

The data analysis revealed significant increases in the standardized mortality ratios among females in 2009 (SMR = 1.25 [1.05–1.48]) and in 2011 (SMR = 1.29 [1.08–1.52]). Increases in the SMRs near statistical significance for females were observed in the years 2012 (SMR = 1.14 [0.96–1.35]), 2013 (SMR = 1.16 [0.97–1.38]), and 2014 (SMR = 1.09 [0.91–1.31]). Among males, no statistically significant increase in the SMR was observed.

Among the total sample, a significantly high SMR was observed in the year 2009 (SMR = 1.21 [1.07–1.37]), and standardized ratios near statistical significance were observed in the years 2013 and 2014. From 2015 to 2021, no significant increases in the SMR were observed.

#### 3.2.2. All Malignant Cancers Mortality

From 2014 to 2021, no significant increases in the SMR were observed.

#### 3.2.3. Lung Cancer Mortality

The highest standardized mortality ratios, which were only nearly statistically significant, were observed among females in 2000, 2011, and 2021. Among males, the highest SMRs, which were only nearly statistically significant, were observed in 2000 and 2009. Among the total sample, the highest SMRs, which were only nearly statistically significant, were observed in 2000 (SMR = 1.37 [0.81–2.21]) and 2009 (SMR = 1.25 [0.72–2.05]). From 2012 to 2021, no increase in the risk of lung cancer was observed.

#### 3.2.4. Colorectal Cancer Mortality

The highest statistically significant standardized mortality ratios for colorectal cancer were observed among females in 1999, 2002, 2009, and 2013. All the observed cases of female colorectal cancer were more than 65 years old. Among the males, no significant SMRs were observed. Among the total sample, only a highly statistically significant SMR was observed in the year 2013 (SMR = 2.00 [1.15–3.28]). The other high SMRs that were only nearly statistically significant were observed in the years 1999, 2002, 2009, 2010, and 2014.

#### 3.2.5. All Non-Malignant Cancer Disease Mortality

The highest statistically significant standardized mortality ratios were observed among the females in the years 2009, 2011, and 2013, and only high standardized ratios near statistical significance were observed in the years 2006, 2010, 2012, 2014, 2015, and 2016. Among the males, a significant SMR was observed only in the year 2006 (SMR = 1.33 [1.08–1.62]). Among the total population, significantly high SMRs were observed in 2009 (SMR = 1.37 [1.18–1.58]), 2010 (SMR = 1.23 [1.06–1.43]), 2011 (SMR = 1.24 [1.06–1.45]), 2012 (SMR = 1.29 [1.10–1.49]), 2013 (SMR = 1.22 [1.05–1.42]), and 2014 (SMR = 1.39 [1.20–1.60]), and only high standardized ratios near statistical significance were observed in the years 2006, 2008, and 2015. From 2016 to 2021, no increase in risk was observed.

#### 3.2.6. All Cardio-Circulatory Disease Mortality

Among the females, only high standardized ratios near statistical significance were observed in 1999, 2007, 2009, 2011, 2014, 2016, and 2020. Among the males, a significant SMR was observed only in the year 2014. Among all the people, a significantly high SMR was observed in the year 2014 (SMR = 1.27 [1.04–1.53]). Only high standardized ratios near statistical significance were observed in 2009 and 2016. The most important analytic causes of these groups of diseases detected in the years where significant SMRs were observed were hypertensive heart diseases [I11] (37–43%) and cerebrovascular diseases [I61–I71] (28–32%).

### 3.3. Mortality Data [SMR’s Temporal Trends]

#### 3.3.1. All Causes of Death

**Figure 1:**
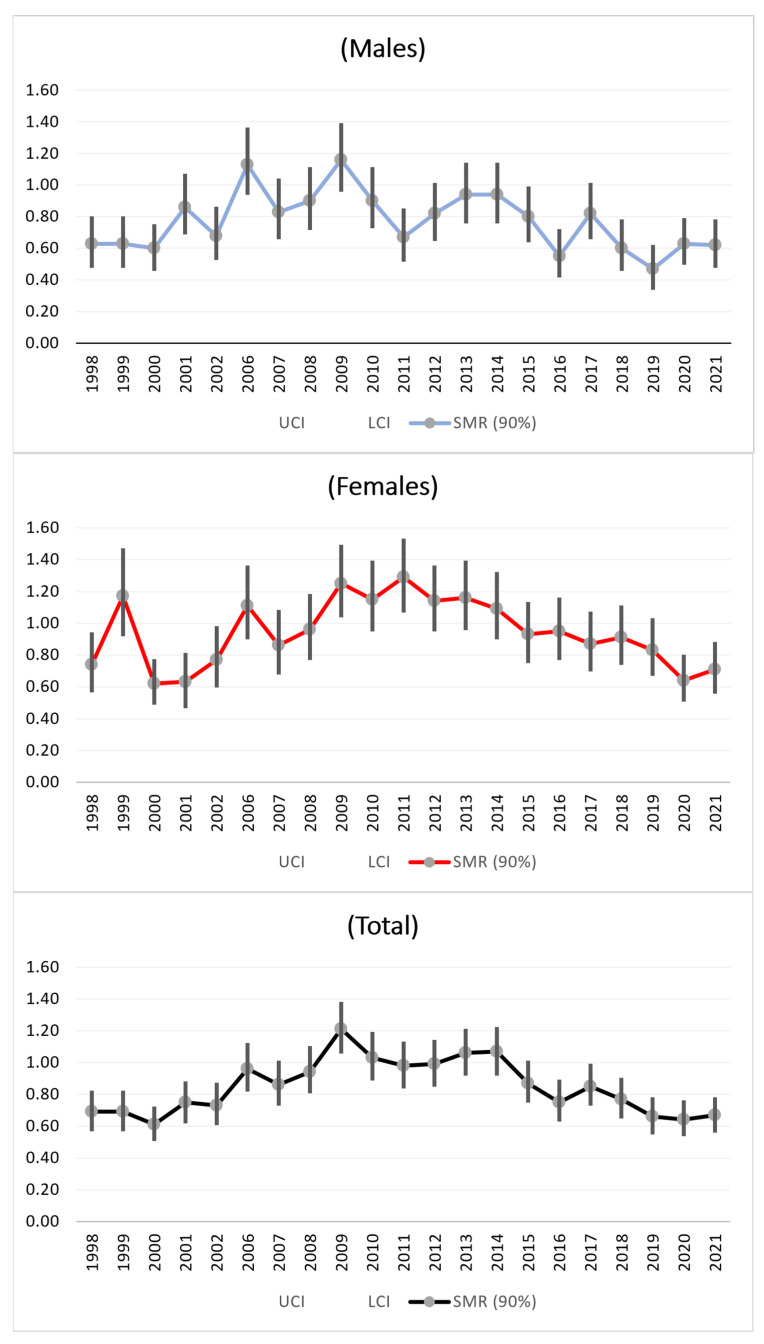
SMRs time trends (1998–2021) of all causes.

From 1998 to 2009, there were increase in mortality from all causes of death among the males, females, and the total population. After 2009, there were decreases in the SMR trends for the total sample, males, and females towards nonsignificant values (Figure 1).

#### 3.3.2. All Malignant Cancers

**Figure 2:**
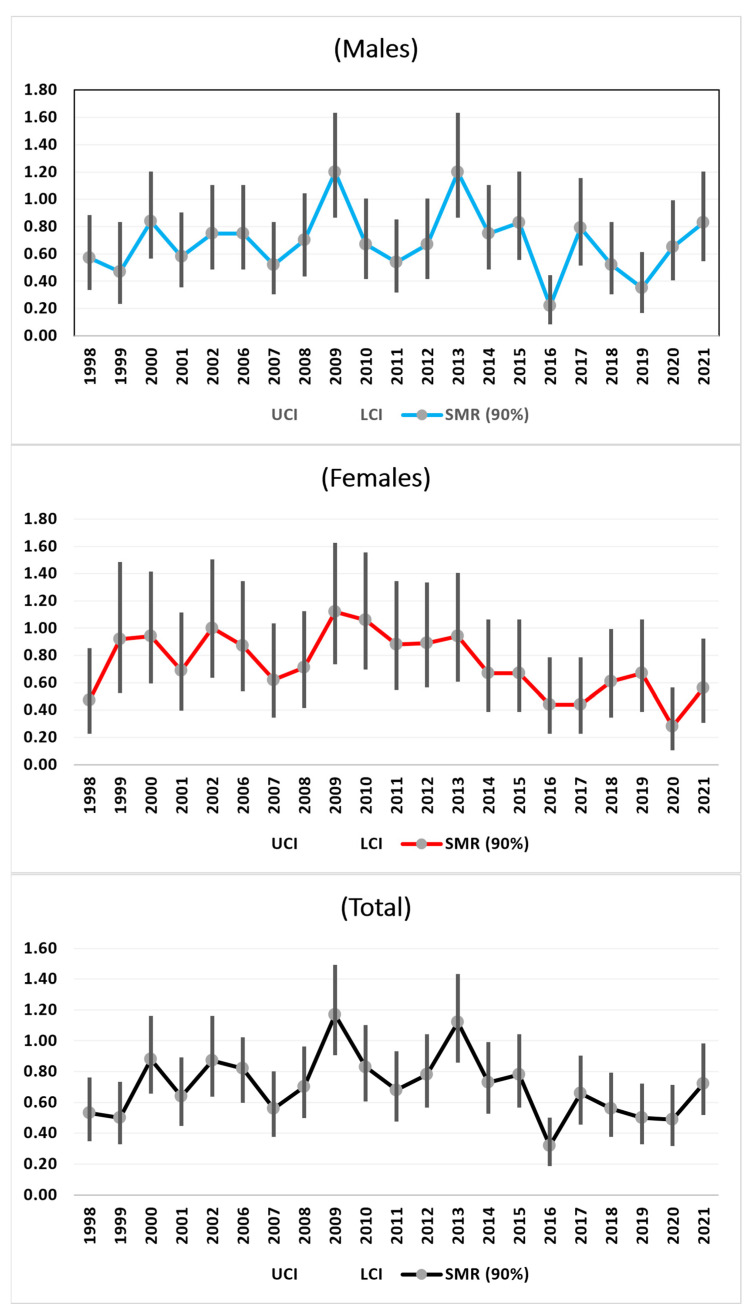
SMRs time trends (1998–2021) of all malignant Cancers.

For malignant cancers, we observed the same increases in the SMR trends from 1998 to 2009 and decreases towards 2020 for the females and total females, with a final increase from 2020 to 2021. For the males, this final increase began in 2019. These final increases were not statistically significant (Figure 2).

#### 3.3.3. Lung Cancer

**Figure 3:**
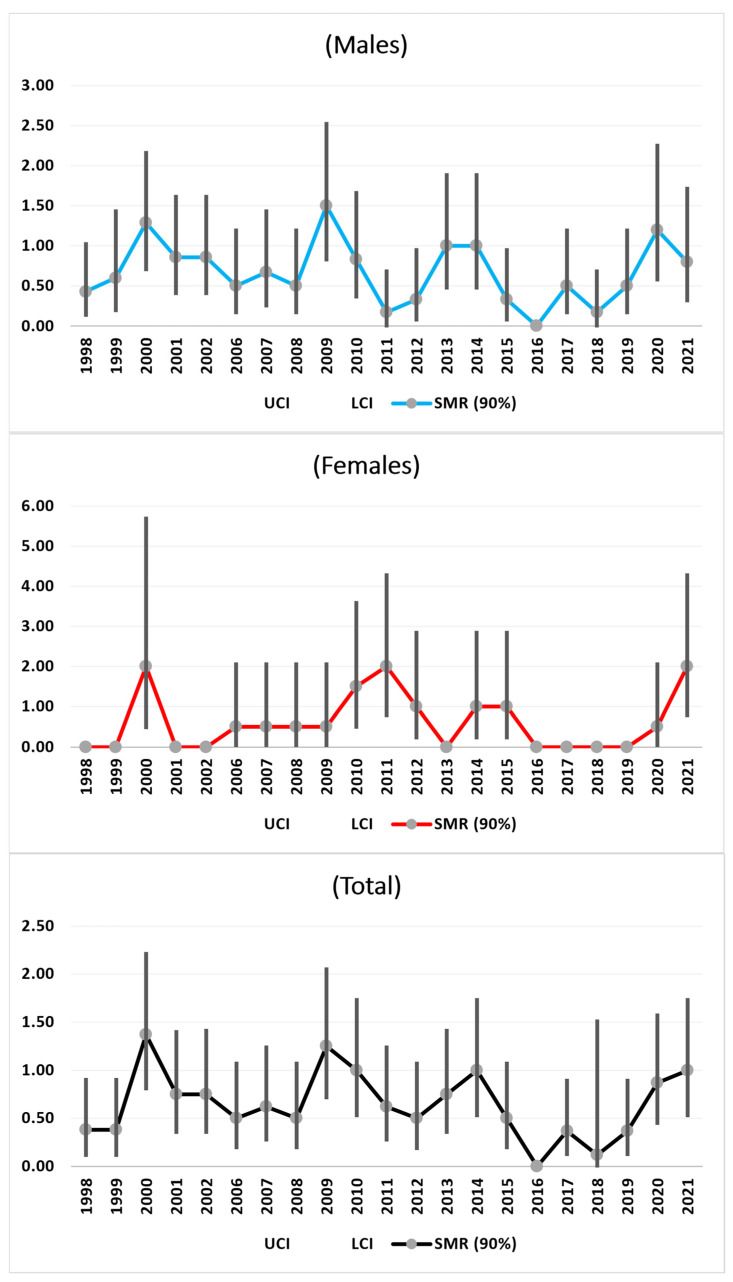
SMRs time trends (1998–2021) of Lung Cancer.

From 1998 to 2009, there were increasing trends in total SMRs, especially among the men. There were decreasing trends until 2018 for both sexes. From 2018 to 2021, a new increase in the total population was observed, especially due to the increase in females, which was very close to statistical significance [SMR = 2.00 (0.79–4.28)] (Figure 3).

#### 3.3.4. Intestinal and Colorectal Cancer

**Figure 4:**
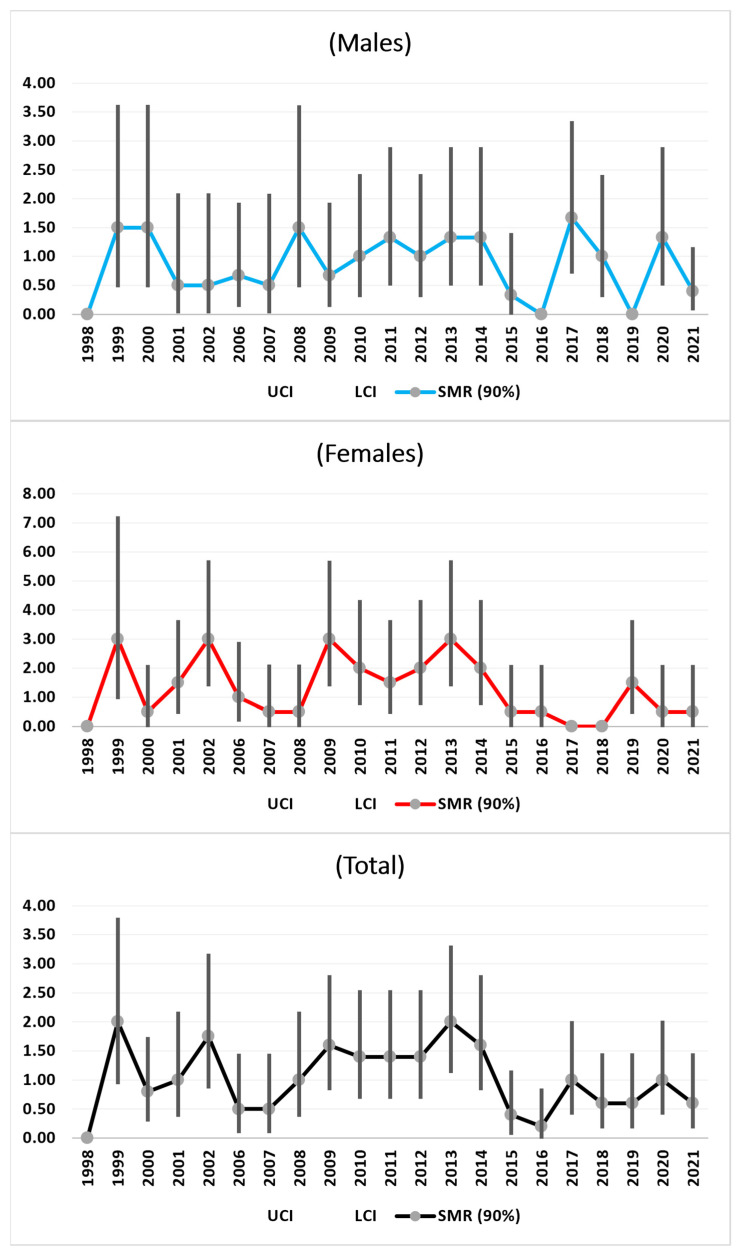
SMRs time trends (1998–2021) of Intestinal & Colon-Rectal Cancer.

There was an increase in the total SMR trend from 1998 to 2013, which was due mainly to the significant SMRs observed among the females. From 2013–2018, there were decreasing trends for the total population and both sexes, although with a high SMR only for the males in 2017. A slight new increase from 2018 to 2021 was also observed (Figure 4).

#### 3.3.5. All Nonmalignant Cancer Diseases

**Figure 5:**
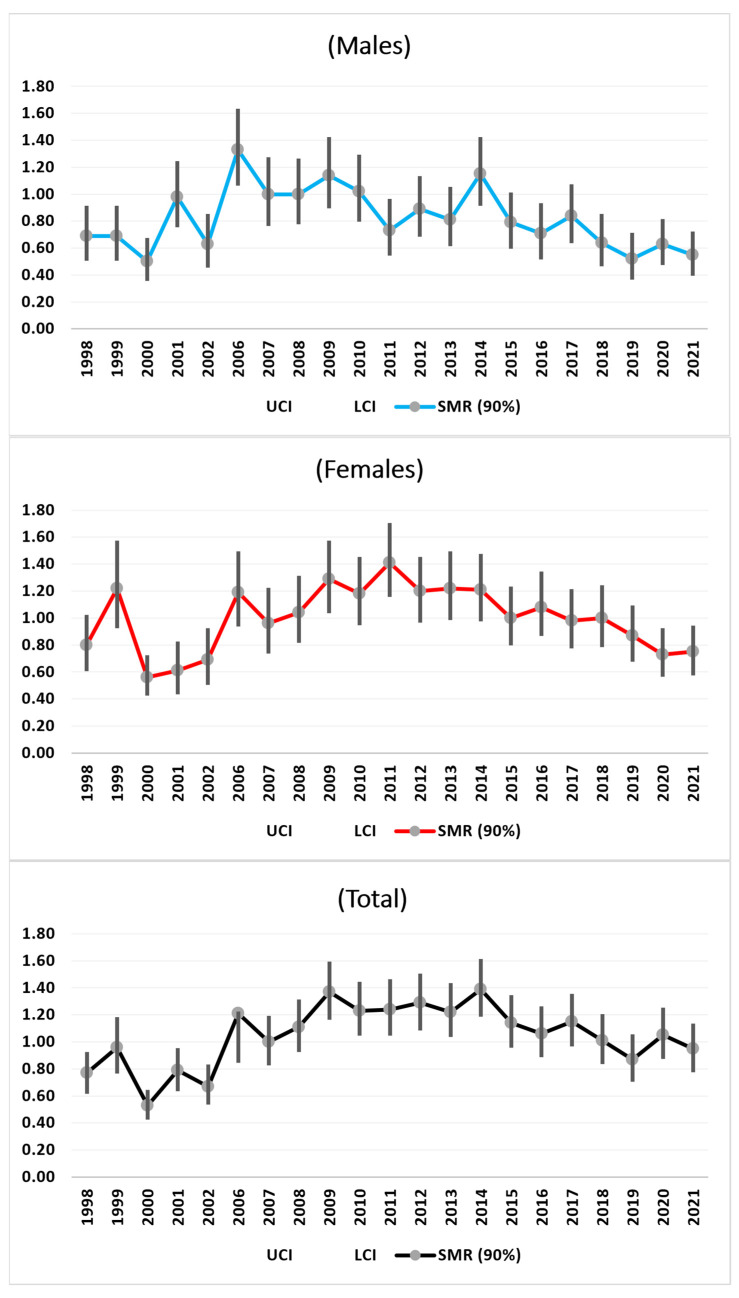
SMRs time trends (1998–2021) of all no-malignant cancer diseases.

An increase in the SMRs for total statistically significant areas was observed from 1998 to 2014, which was due mainly to the increase in the females. From 2014 to 2021, there was a substantial decrease in the trend in nonsignificant SMRs (Figure 5).

#### 3.3.6. Circulatory System and Cardiovascular Diseases

**Figure 6:**
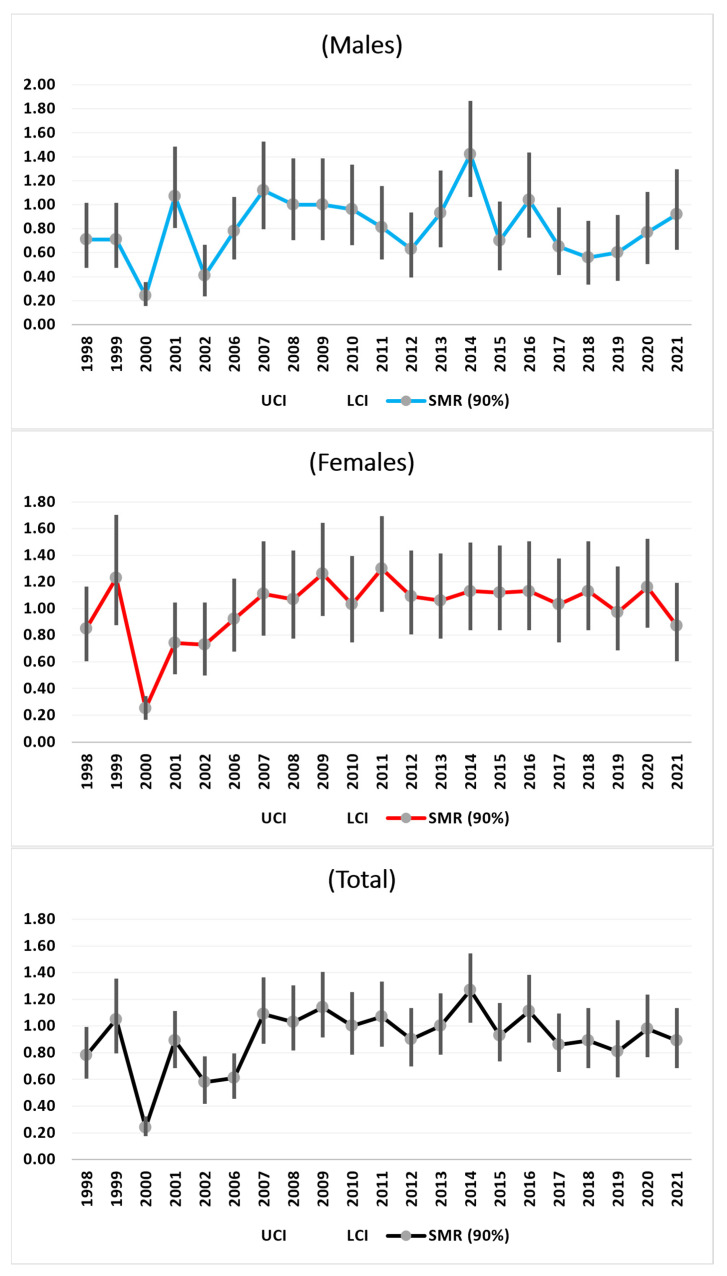
SMRs time trends (1998–2021) of cardiovascular diseases.

The observed trends of the SMRs increased from 1998 to 2007 because of both sexes but was due mainly to the females. From 2007 to 2021, a stable trend in total SMRs was revealed, which was influenced mainly by the female SMR, which was generally nearly statistically significant. Only in 2014 did the male SMR influence the total SMR, and it was also statistically significant. After this period, a decreasing stable trend was observed (Figure 6).

## 4. Discussion

The results of this mortality study revealed, among the females, significantly high standardized mortality ratios (SMRs) for all causes in 2009 (Figure 1). After 2009, a significant decrease in the SMR for all-cause mortality was evident. These results are compatible with the results of a study from 2014 that showed that, in Italy, a consistent decline in all-cause mortality occurred across the whole period (1901–2008), with the most striking variations being observed in the 0–49-year-old population. In 2008, the main causes of death were accidents (males) and tumors (females) in the 0–49 years age class, tumors in the 50–69 years age class (both sexes), and tumors (males) and cardiovascular diseases (females) in elderly individuals. The results highlight the potential interplay between age and sex in affecting mortality trends and reflect dramatic progress in nutritional, lifestyle, socioeconomic, medical, and hygienic conditions [27].

Mortality from all cancers and the most common cancer sites has declined over the last 25 years, except for the pancreas and lung (in women) in many countries [1]. Apulian digestive system tumor rates are aligned with the rates in Southern Italy, whereas lung cancer rates in men are aligned with overall Italian rates; in contrast, in women, lung cancer rates are aligned with the rates in Southern Italy. In addition, we found a very similar ranking of cancer between Apulia and Italy; the first five items are prostate, lung, colon–rectum, urinary bladder, and stomach in Italian men. Liver cancer in Apulia replaces head and neck cancer. Among women, the ranking of cancer incidence is breast, colorectal, thyroid, lung, and corpus uteri in Italian women and breast, colorectal, thyroid, corpus uteri, and lung in Apulian women. Compared with Italian women, Apulian women are more likely to have lung cancer [28,29].

For the assessment of radon exposure and cancer mortality/incidence risk, strong implementation of occupational cancer epidemiology is necessary [30]. The study of residential radon exposure and cancer is also at the center of scientific research [31].

In this study, only after 2009 did the trend of SMRs for lung cancer decrease, which is similar to the trend described by the Independent High-level Commission on Noncommunicable Diseases (NCD) of the World Health Organization (WHO). In this work, a global reduction in lung cancer mortality has been reported since the year 2000, although this effect is not sufficient to reach the 30% reduction in mortality from NCDs by the year 2030, as stipulated by the United Nations Sustainable Development Goal 3.4. Our data from 1998 to 2009 are also different from those of an Apulian study related to the period from 1933 to 2010, in which TSDs for respiratory tumors and bronchitis decreased in all areas analyzed. However, in the provinces of Taranto, Brindisi, and Lecce, the SMR for respiratory tumors was lower than the national reference until the 1960s, which aligns (in Brindisi) and exceeds (in Lecce and Taranto) the reference in subsequent years. In the provinces of Foggia and Bari, the number of deaths from lung cancer is consistently lower than expected [32]. This is true for our data only after 2009 until 2019.

The observed increasing trends in the total SMRs for intestinal and colon cancers until 2013 were substantially due to female trends (Figure 4). These findings are compatible with data from the Global Health Observatory of the World Health Organization, which reported that colorectal cancer is the third most common cancer in the world and is one of the most common causes of female cancer mortality after breast cancer. The incidence and mortality of colorectal cancer in populations over 65 years of age are greater in women than in men, implying that colorectal cancer is a major health threat among older women [33]. Additionally, long-term trends in Europe over three decades have been published [34]. A recent systemic review reported that a greater proportion of women presented higher right-sided colon cancer mortality than men did. Patients with right-sided colon cancer are often at a more advanced stage at diagnosis [35]. In women, the five leading primary cancer sites are the breast, colorectum, lung, thyroid, and stomach, whereas lung, colorectal, pancreatic, liver, and breast cancers are projected to be the most common causes of cancer-related death [36].

Some studies have shown that in women, total fat intake (OR = 1.9) and high caloric intake (OR = 1.5) are associated with increased colon cancer risk after adjusting for age, body mass index, and crude fiber [37].

General occupational and nonoccupational natural radiation (radon) exposure is indicated in some studies [38,39,40]. Radon in groundwater has been associated with lung and stomach cancer, but to our knowledge, there is also little evidence for an association between radon and colorectal cancer (CRC) [41]. Residential radon exposure and nonlung cancer incidence/mortality were also reported in a study conducted in Galicia [42].

There are different possible causes of CRC. First, women possess a longer transverse colon than men do, resulting in a lower detection rate in colonoscopy [43]. This characteristic is also linked to reductions in the sensitivity and specificity of screening tests for females, resulting in a reduction in their survival [44]. Moreover, the sensitivity of the fecal occult blood test (FEBT) differs by sex, and the lower specificity and negative predictive value among females can explain their higher mortality and lower survival for CRC [45]. Despite sex-specific differences in dietary risk factors associated with cancer risk, the evidence for generating sex-specific summary estimates is limited. The possible associations between the socioeconomic circumstances of women and cancer treatment also require attention [4]. The association between a “dietary inflammatory index” and CRC has also been studied [46]. Additionally, an association between dairy product consumption and CRC in the older Mediterranean population was observed [47]. Hormonal factors may explain a large percentage of right-sided colorectal cancer cases in females. A population-based case-control study examining sex, reproductive factors, and hormone exposure related to microsatellite instability (MSI) in patients with colon cancer (n = 4246) suggested that estrogen exposure is a protective factor against MSI, whereas a lack of estrogen in older women increased the risk of MSI-high colon cancer [5]. Nongenetic determinants for early-onset CRC have also been studied [48]. BRCA1 and BRCA2 genetic mutation carriers and the incidence of CRC were also studied [49]. The ESR2 protein expression levels in CRC have also been studied [50]. Mutation of the KRAS and MMR genes in a series of CRC cases was also observed [51]. Body size and CRC risk were also evaluated in postmenopausal women [52,53]. New methods for the assessment of CRC incidence rates based on structural equation modeling are now available, and we hope that in the future, it will be possible to apply these methods to mortality data [54]. Additionally, new methods for the identification of metabolic features of CRC liability are available [55].

The significant increase in nonmalignant cancer SMRs among females from 1998 to 2014 and the nonsignificant decrease (Figure 5) were due mainly to circulatory, respiratory, and digestive system diseases. These results are similar to those of several international studies in which the observed reductions in respiratory and dementia deaths and the reduced seasonality in ischemic heart disease deaths may reflect reductions in circulating respiratory (non-SARS-CoV-2) pathogens resulting from the public health measures taken in 2020 [56]. In contrast, in much of Latin America, mortality from respiratory disease gradually decreased between 1998 and 2008. However, this downwards trend halted in 2009, likely as a result of the (H1N1) 2009 pandemic [57].

With respect to circulatory system diseases, the increased observed SMR trends until 2014 and their stability from 2014 to 2021 were similar to those reported in other national and international studies where ischemic heart disease (IHD) was the single leading cause of death. Rates are different across countries and are decreasing in most countries, indicating great potential for further gains. In the future, improvements in these decreases may be curtailed by the increasing incidence of hypertension in some developing countries and, more importantly, the global growth of obesity [58]. Frailty increases cardiovascular (CV) morbidity and mortality in patients both with and without known CV disease. Although the recognition of this additional risk factor has become increasingly clinically relevant in CV diseases, uncertainty remains regarding operative definitions, screening, assessment, and management of frailty [59]. Atrial fibrillation (AF) is associated with many circulatory diseases. Compared with non-AF-associated deaths, the strongest associations were observed between AF and hypertensive diseases (prevalence ratio 1.62, 95% CI 1.57–1.67), cardiac valve disorders (2.43, 2.25–2.61), cardiomyopathies (1.93, 1.70–2.19), cerebrovascular diseases (1.55, 1.50–1.60), and chronic obstructive pulmonary disease (1.49, 1.42–1.57). AF-associated mortality was higher than previously reported, probably due to the ageing of the population with multiple predisposing diseases, increased recognition of AF among elderly individuals, and increased awareness of certifying physicians about the importance of AF [60].

The associations between the exposure of workers and the general population to radon and cerebrovascular diseases were described in a very important meta-analysis [61]. In 2015, age-standardized ischemic heart disease (IHD) death rates in Eastern European and Central Asian countries were almost two times greater than those of satellite states in Central Europe. Between 1990 and 2015, rates decreased substantially in Central Europe (men −43.5% (95% uncertainty interval −45.0%, −42.0%); women −42.9% (−44.0%, −41.0%)) but less in Eastern Europe (men −5.6% (−9.0, –3.0); women −12.2% (−15.5%, −9.0%)). Age-standardized IHD death rates also varied within regions: within Eastern Europe, rates decreased by −51.7% in Estonian men (−54.0, −47.0) but increased by +19.4% in Belarusian men (+12.0, +27.0). High blood pressure and cholesterol are leading risk factors for IHD burden, with smoking, body mass index, dietary factors, and ambient air pollution also ranking high [62]. Among U.S. young adults, the prevalence rates of hypercholesterolemia, hypertension, and diabetes were 8.8% (SE = 0.4%), 7.3% (SE = 0.3%), and 2.6% (SE = 0.2%), respectively. The prevalence rates of borderline high cholesterol, blood pressure, and blood glucose were substantially greater (21.6% [SE = 0.6%], 26.9% [SE = 0.7%], and 18.9% [SE = 0.6%], respectively) [63]. In Brazil, cardiovascular disease (CVD) has been the leading cause of mortality since the 1960s and accounts for a substantial percentage of all hospitalizations. In 2011, CVD was responsible for 31% of all deaths, with ischemic heart disease (31%) and cerebrovascular diseases (30%) being the leading causes of CVD. Despite an increase in the overall number of CVD deaths, the age-adjusted mortality rates for CVD declined by 24% between 2000 and 2011 [64].

This study has several limitations. First, it was not possible to acquire data related to the three-year period of 2003–2005 because the death records were stored in another location, and we did not have permission to access them. Nevertheless, the absence of these data is not substantially important for the structure of the study results. Another bias is the quality of the dataset of the local health authority (ASL), which was not possible to verify.

However, despite these limitations, this study provides only a small piece of evidence in support of previous scientific studies and needs further in-depth verification through the performance of appropriate multiple-measure and multidisciplinary long-term studies to increase the number of appropriate preventive measures. In particular, a new future study is needed to assess the associations between observed lung cancer cases and radon domestic exposure to drive radon mitigation and public health strategies. This result deserves attention because residential and occupational radon levels can usually be reduced, often through simple and low-cost intervention measures, with the prospect of reducing the number of radon-related lung cancer deaths through preventive measures.

## 5. Conclusions

Radon is a known risk factor for lung cancer, and residential radon exposure is the leading cause of lung cancer in never-smokers; however, in Italy, there is still a lack of public awareness regarding the risk caused by residential radon exposure.

This mortality study (1st step) is important for the quantification of lung cancer deaths and the identification of their home addresses for the planning of a new case-control study (2nd step) to assess the association between domestic environmental radon measurements with new sensors and lung cancer cases and then compare them with the measurements carried out in the homes of randomly selected controls. Identifying an association between radon exposure and the mortality rate will be useful for strengthening the estimates reported in previous studies to drive radon mitigation and public health strategies [20].

## Figures and Tables

**Table 1:** Tabulated values of 95% confidence limit factors for a Poisson-distributed variable.

Observed No. of Events on Wich Estimates Is Based	Lower Limit Factor	Upper Limit Factor	Observed No. of Events on Wich Estimates Is Based	Lower Limit Factor	Upper Limit Factor	Observed No. of Events on Wich Estimates Is Based	Lower Limit Factor	Upper Limit Factor
1	0.025	5.57	21	0.619	1.53	120	0.833	1.20
2	0.121	3.61	22	0.627	1.51	140	0.844	1.18
3	0.206	2.92	23	0.634	1.50	160	0.854	1.17
4	0.272	2.56	24	0.641	1.49	180	0.862	1.16
5	0.324	2.33	25	0.647	1.48	200	0.868	1.15
6	0.367	2.18	26	0.653	1.47	250	0.882	1.13
7	0.401	2.06	27	0.659	1.46	300	0.892	1.12
8	0.431	1.97	28	0.665	1.45	350	0.899	1.11
9	0.458	1.90	29	0.670	1.44	400	0.906	1.10
10	0.480	1.84	30	0.675	1.43	450	0.911	1.10
11	0.499	1.79	35	0.697	1.39	500	0.915	1.09
12	0.517	1.75	40	0.714	1.36	600	0.922	1.08
13	0.532	1.71	45	0.729	1.34	700	0.928	1.08
14	0.546	1.68	50	0.742	1.32	800	0.932	1.07
15	0.560	1.65	60	0.770	1.30	900	0.936	1.07
16	0.572	1.62	70	0.785	1.27	1000	0.939	1.06
17	0.583	1.60	80	0.798	1.25			
18	0.593	1.58	90	0.809	1.24			
19	0.602	1.56	100	0.818	1.22			
20	0.611	1.54						

**Table 2:** Data of the study population in the period 2002–2021 (ISTAT source).

	2002								2021							
Age Class	Unmarried	Married	Widowers	Divorced	Males	Females	Total		Unmarried	Married	Widowers	Divorced	Males	Females	Total	
							n	%							n	%
0–4	609	0	0	0	329 (54.00%)	280 (46.00%)	609	4.40%	535	0	0	0	285 (53.30%)	250 (46.70%)	535	3.80%
5–9	730	0	0	0	386 (52.90%)	344 (47.10%)	730	5.20%	573	0	0	0	315 (55.00%)	258 (45.00%)	573	4.10%
10–14	795	0	0	0	405 (50.90%)	390 (40.10%)	795	5.70%	615	0	0	0	318 (51.70%)	297 (48.30%)	615	4.40%
15–19	853	3	0	0	445 (52.00%)	411 (48.00%)	856	6.10%	655	0	0	0	340 (51.90%)	315 (48.10%)	655	4.70%
20–24	868	80	0	0	471 (49.70%)	477 (50.305)	948	6.80%	623	11	0	0	350 (55.20%)	284 (44.80%)	634	4.50%
25–29	661	341	1	0	501 (50.00%)	502 (50.00%)	1.003	7.20%	648	73	1	4	363 (50.00%)	363 (50.00%)	726	5.20%
30–34	347	683	1	3	511 (49.40%)	523 (50.60%)	1.034	7.40%	529	275	1	6	396 (48.80%)	415 (51.20%)	811	5.80%
35–39	199	887	5	13	506 (45.80%)	598 (54.20%)	1.104	7.90%	389	491	1	20	432 (47.90%)	469 (52.10%)	901	6.40%
40–44	97	933	9	13	505 (48.00%)	547 (52.00%)	1.052	7.60%	308	662	2	32	508 (50.60%)	496 (49.40%)	1.004	7.10%
45–49	64	786	20	11	450 (51.10%)	431 (48.90%)	881	6.30%	234	759	20	48	522 (49.20%)	539 (50.80%)	1.061	7.50%
50–54	52	734	36	12	411 (49.30%)	423 (50.70%)	834	6.00%	156	796	22	58	491 (47.60%)	541 (52.40%)	1.032	7.30%
55–59	55	591	31	9	331 (48.30%)	355 (51.70%)	686	4.90%	129	932	37	61	533 (46.00%)	626 (54.00%)	1.159	8.20%
60–64	41	597	60	7	332 (47.10%)	373 (52.90%)	705	5.10%	88	851	43	39	488 (47.80%)	533 (52.20)	1.021	7.30%
65–69	70	569	117	7	329 (43.10%)	434 (56.90%)	763	5.50%	50	690	76	28	417 (49.40%)	427 (50.60%)	844	6.00%
70–74	66	489	167	4	325 (44.80%)	401 (55.20%)	726	5.20%	39	580	123	29	373 (48.40%)	398 (51.60%)	771	5.50%
75–79	56	355	188	4	274 (45.40%)	329 (54.60%)	603	4.30%	34	387	136	16	263 (45.90%)	310 (54.10%)	573	4.10%
80–84	26	153	126	0	112 (36.70%)	193 (63.30%)	305	2.20%	34	333	175	6	243 (44.30%)	305 (55.70%)	548	3.90%
85–89	15	74	118	0	86 (41.50%)	121 (58.50%)	207	1.50%	25	152	216	2	145 (36.70%)	250 (63.30%)	395	2.80%
90–94	5	7	57	1	17 (24.30%)	53 (75.70%)	70	0.50%	9	58	97	1	60 (36.40%)	105 (63.60%)	165	1.20%
95–99	1	2	13	0	6 (37.50%)	10 (62.50%)	16	0.10%	0	4	33	0	9 (24.30%)	28 (75.70%)	37	0.30%
100+	0	0	0	0	0 (00.00%)	0 (00.00%)	0	0.00%	3	0	3	0	0 (00.00%)	6 (100.00%)	6	0.00%
Total	5.610	7.284	949	84	6732 (48.30%)	7195 (51.70%)	13.927	100.00%	5.676	7.054	986	350	6851 (48.70%)	7215 (51.30%)	14.066	100.00%

## Data Availability

The raw data supporting the conclusions of this article will be made available by the authors on request.

## References

[1] Santucci C., Carioli G., Bertuccio P., Malvezzi M., Pastorino U., Boffetta P., Negri E., Bosetti C., La Vecchia C. (2020). Progress in cancer mortality, incidence, and survival: A global overview. Eur. J. Cancer Prev..

[2] Ferlay J., Ervik M., Lam F., Laversanne M., Colombet M., Mery L., Piñeros M., Znaor A., Soerjomataram I., Bray F. (2024). Global Cancer Observatory: Cancer Today.

[3] Bade B.C., Dela Cruz C.S. (2020). Lung Cancer 2020: Epidemiology, Etiology, and Prevention. Clin. Chest Med..

[4] Kim S.E., Paik H.Y., Yoon H., Lee J.E., Kim N., Sung M.K. (2015). Sex and gender-specific disparities in colorectal cancer risk. World J. Gastroenterol..

[5] Slattery M.L., Potter J.D., Curtin K., Edwards S., Ma K.N., Anderson K., Schaffer D., Samowitz W.S. (2001). Estrogens reduce and withdrawal of estrogens increase risk of microsatellite instability-positive colon cancer. Cancer Res..

[6] Bae J.M., Kim J.H., Cho N.Y., Kim T.Y., Kang G.H. (2013). Prognostic implication of the CpG island methylator phenotype in colorectal cancers depends on tumor location. Br. J. Cancer.

[7] Wiencke J.K., Zheng S., Lafuente A., Lafuente M.J., Grudzen C., Wrensch M.R., Miike R., Ballesta A., Trias M. (1999). Aberrant methylation of p16INK4a in anatomic and gender-specific subtypes of sporadic colorectal cancer. Cancer Epidemiol. Biomark. Prev..

[8] Bae S.J., Kim J.W., Kang H., Hwang S.G., Oh D., Kim N.K. (2008). Gender specific association between polymorphism of vascular endothelial growth factor (VEGF 936 C>T) gene and colon cancer in Korea. Anticancer Res..

[9] Wardle J., Haase A.M., Steptoe A., Nillapun M., Jonwutiwes K., Bellisle F. (2004). Gender differences in food choice: The contribution of health beliefs and dieting. Ann. Behav. Med..

[10] Kaku E., Oda Y., Murakami Y., Goto H., Tanaka T., Hasuda K., Yasunaga M., Ito K., Sakurai K., Fujimori T. (2011). Proportion of flatand depressed-type and laterally spreading tumor among advanced colorectal neoplasia. Clin. Gastroenterol. Hepatol..

[11] O’Keefe S.J. (2016). Diet, microorganisms and their metabolites, and colon cancer. Nat. Rev. Gastroenterol. Hepatol..

[12] Bultman S.J. (2017). Interplay between diet, gut microbiota, epigenetic events, and colorectal cancer. Mol. Nutr. Food Res..

[13] Huang J., Fang Y.J., Xu M., Luo H., Zhang N.Q., Huang W.Q., Pan Z.Z., Chen Y.M., Zhang C.X. (2018). Carbohydrate, dietary glycaemic index and glycaemic load, and colorectal cancer risk: A case-control study in China. Br. J. Nutr..

[14] Hu J., La Vecchia C., Negri E., Mery L. (2010). Nutrients and risk of colon cancer. Cancers.

[15] Ferrucci L.M., Sinha R., Huang W.Y., Berndt S.I., Katki H.A., Schoen R.E., Hayes R.B., Cross A.J. (2012). Meat consumption and the risk of incident distal colon and rectal adenoma. Br. J. Cancer.

[16] Stemmermann G.N., Nomura A., Chyou P.H. (1990). The influence of dairy and nondairy calcium on subsite large-bowel cancer risk. Dis. Colon Rectum.

[17] Wu K., Willett W.C., Fuchs C.S., Colditz G.A., Giovannucci E.L. (2002). Calcium intake and risk of colon cancer in women and men. J. Natl. Cancer Inst..

[18] Oh K., Willett W.C., Wu K., Fuchs C.S., Giovannucci E.L. (2007). Calcium and vitamin D intakes in relation to risk of distal colorectal adenoma in women. Am. J. Epidemiol..

[19] Barber L.E., McCullough L.E., Johnson D.A. (2024). Eyes Wide Open: Sleep as a Potential Contributor to Racial and Ethnic Disparities in Cancer. Cancer Epidemiol. Biomark. Prev..

[20] Ferri G.M., Intranuovo G., Cavone D., Corrado V., Birtolo F., Tricase P., Fuso R., Vilardi V., Sumerano M., L’abbate N. (2018). Estimates of the Lung Cancer Cases Attributable to Radon in Municipalities of Two Apulia Provinces (Italy) and Assessment of Main Exposure Determinants. Int. J. Environ. Res. Public Health.

[21] Vimercati L., Cavone D., Delfino M.C., De Maria L., Caputi A., Sponselli S., Corrado V., Bruno V., Spalluto G., Eranio G. (2021). Relationships among Indoor Radon, Earthquake Magnitude Data and Lung Cancer Risks in a Residential Building of an Apulian Town (Southern Italy). Atmosphere.

[22] Haenszel W. (1969). Report of the working group on studies of cancer and related diseases in migrant populations. Int. J. Cancer.

[23] Breslow N.E., Day N.E. Statistical Methods in Cancer Research. Vol. II. The Design and Analysis of Cohort Studies. https://publications.iarc.fr/Book-And-Report-Series/Iarc-Scientific-Publications/Statistical-Methods-In-Cancer-Research-Volume-II-The-Design-And-Analysis-Of-Cohort-Studies-1986.

[24] Haenszel W., Loveland D.B., Sirken M.G. (1962). Lung-cancer mortality as related to residence and smoking histories. I. White males. J. Natl. Cancer Inst..

[25] Rothman K.J., Boice J.D. (1979). Epidemiologic Analysis with a Programmable Calculator.

[26] Sullivan K.M., Dean A., Soe M.M. (2009). OpenEpi: A Web-based Epidemiologic and Statistical Calculator for Public Health. Public Health Rep..

[27] Vercelli M., Lillini R., Quaglia A., Micale R.T., La Maestra S., De Flora S. (2014). Age-Related Mortality Trends in Italy from 1901 to 2008. PLoS ONE.

[28] Nannavecchia A.M., Rashid I., Cuccaro F., Chieti A., Bruno D., Burgio Lo Monaco M.G., Tanzarella C., Bisceglia L. (2017). Cancer incidence estimation method: An Apulian experience. Eur. J. Cancer Prev..

[29] Rashid I., Cozza V., Bisceglia L. (2024). Cancer figures in Italy: An overview. Epidemiol. Prev..

[30] Turner M.C. (2022). What is next for occupational cancer epidemiology?. Scand J. Work. Environ. Health.

[31] Reddy A., Conde C., Peterson C., Nugent K. (2022). Residential radon exposure and cancer. Oncol. Rev..

[32] Montinari M.R., Gianicolo E.A.L., Vigotti M.A. (2016). Mortality from respiratory diseases in the provinces of Apulia Region (Southern Italy) from 1933 to 2010. Epidemiol. Prev..

[33] Ferlay J., Soerjomataram I., Ervik M., Dikshit R., Eser S., Mathers C., Rebelo M., Parkin D.M., Forman D., Bray F. (2012). GLOBOCAN 2012 Estimated: Cancer Incidence and Mortality Worldwide in 2012 v1.0.

[34] Long D., Mao C., Zhang Z., Liu Y., Li J., Xu Y., Zhu Y. (2023). Long-term trends in the burden of colorectal cancer in Europe over three decades: A join point regression and age-period cohort analysis. Front. Oncol..

[35] Hansen I.O., Jess P. (2012). Possible better long-term survival in left versus right-sided colon cancer—A systematic review. Dan. Med. J..

[36] Jung K.W., Won M.S.Y., Hong S., Kong H.J., Lee E.S. (2020). Prediction of Cancer Incidence and Mortality in Korea, 2020. Cancer Res. Treat..

[37] West D.W., Slattery M.L., Robison L.M., Schuman K.L., Ford M.H., Mahoney A.W., Lyon J.L., Sorensen A.W. (1989). Dietary intake and colon cancer: Sex- and anatomic site-specific associations. Am. J. Epidemiol..

[38] Missimer M., Teaf C., Maliva R.G., Danley-Thomson A., Covert D., Hegy M. (2019). Natural Radiation in the Rocks, Soils, and Groundwater of Southern Florida with a Discussion on Potential Health Impacts. Int. J. Environ. Res. Public Health.

[39] Vimercati L., Fucilli F., Cavone D., De Maria L., Birtolo F., Ferri G.M., Soleo L., Lovreglio P. (2018). Radon Levels in Indoor Environments of the University Hospital in Bari-Apulia Region Southern Italy. Int. J. Environ. Res. Public Health.

[40] De Maria L., Sponselli S., Caputi A., Delvecchio G., Giannelli G., Pipoli A., Cafaro F., Zagaria S., Cavone D., Sardone R. (2023). Indoor Radon Concentration Levels in Healthcare Settings: The Results of an Environmental Monitoring in a Large Italian University Hospital. Int. J. Environ. Res. Public Health.

[41] Messier K.P., Serre M.L. (2017). Lung and stomach cancer associations with groundwater radon in North Carolina, USA. Int. J. Epidemiol..

[42] Barbosa-Lorenzo R., Barros-Dios J.M., Raìces Aldrey M., Cerdeira Caramés S., Ruano-Ravina A. (2016). Residential radon and cancers other than lung cancer: A cohort study in Galicia, a Spanish radon-prone area. Eur. J. Epidemiol..

[43] Saunders B.P., Fukumoto M., Halligan S., Jobling C., Moussa M.E., Bartram C.I., Williams C.B. (1996). Why is colonoscopy more difficult in women?. Gastrointest. Endosc..

[44] Hur H., Oh C.M., Won Y.J., Oh J.H., Kim N.K. (2018). Characteristics and Survival of Korean Patients with Colorectal Cancer Based on Data from the Korea Central Cancer Registry Data. Ann. Coloproctol..

[45] Brenner H., Haug U., Hundt S. (2010). Sex differences in performance of fecal occult blood testing. Am. J. Gastroenterol..

[46] Cho Y.A., Lee J., Oh J.H., Shin A., Kim J. (2016). Dietary Inflammatory Index and Risk of Colorectal Cancer: A Case-Control Study in Korea. Nutrients.

[47] Barrubés L., Babio N., Mena-Sánchez G., Toledo E., Ramírez-Sabio J.B., Estruch R., Ros E., Fitó M., Arós F., Fiol M. (2018). Dairy product consumption and risk of colorectal cancer in an older mediterranean population at high cardiovascular risk. Int. J. Cancer.

[48] Archambault A.N., Lin Y., Jeon J., Harrison T.A., Bishop D.T., Brenner H., Casey G., Chan A.T., Chang-Claude J., Figueiredo J.C. (2021). Nongenetic Determinants of Risk for Early-Onset Colorectal Cancer. JNCI Cancer Spectr..

[49] Phelan C.M., Iqbal J., Lynch H.T., Lubinski J., Gronwald J., Moller P., Ghadirian P., Foulkes W.D., Armel S., Eisen A. (2014). Incidence of colorectal cancer in BRCA1 and BRCA2 mutation carriers: Results from a follow-up study. Br. J. Cancer.

[50] Tillmans L.S., Vierkant R.A., Wang A.H., Samadder N.J., Lynch C.F., Anderson K.E., French A.J., Haile R.W., Harnack L.J., Potter J.D. (2015). Associations between Environmental Exposures and Incident Colorectal Cancer by ESR2 Protein Expression Level in a Population-Based Cohort of Older Women. Cancer Epidemiol. Biomark. Prev..

[51] Cionca F.L., Dobre M., Dobrea C.M., Iosif C.I., Comănescu M.V., Ardeleanu C.M. (2018). Mutational status of KRAS and MMR genes in a series of colorectal carcinoma cases. Rom. J. Morphol. Embryol..

[52] Su L., Hendryx M., Li M., Shadyab A.H., Saquib N., Stefanick M.L., Luo J. (2023). Body size over the adult life course and the risk of colorectal cancer among postmenopausal women. Public Health Nutr..

[53] Oxentenko A.S., Bardia A., Vierkant R.A., Wang A.H., Anderson K.E., Campbell P.T., Sellers T.A., Folsom A.R., Cerhan J.R., Limburg P.J. (2010). Body size and incident colorectal cancer: A prospective study of older women. Cancer Prev. Res..

[54] Schwartz G.G., Klug M.G., Rundquist B.C. (2019). An exploration of colorectal cancer incidence rates in North Dakota, USA, via structural equation modeling. Int. J. Color. Dis..

[55] Bull C., Hazelwood E., Bell J.A., Tan V., Constantinescu A.E., Borges C., Legge D., Burrows K., Huyghe J.R., Brenner H. (2023). Identifying metabolic features of colorectal cancer liability using Mendelian randomization. eLife.

[56] Gregory G., Zhu L., Hayen A., Bell K.J.L. (2022). Learning from the pandemic: Mortality trends and seasonality of deaths in Australia in 2020. Int. J. Epidemiol..

[57] de Souza M.D.F.M., Widdowson M.A., Alencar A.P., Gawryszewski V.P., Aziz-Baumgartner E., Palekar R., Breese J., Cheng P.Y., Barbosa J., Cabrera A.M. (2013). Trends in mortality from respiratory disease in Latin America since 1998 and the impact of the 2009 influenza pandemic. Bull. World Health Organ..

[58] Nowbar A.N., Gitto M., Howard J.P., Francis D.P., Al-Lamee R. (2019). Mortality From. Ischemic Heart Disease Analysis of Data from the World Health Organization and Coronary Artery Disease Risk Factors from NCD Risk Factor Collaboration. Circ. Cardiovasc. Qual. Outcomes.

[59] Richter D., Guasti L., Walker D., Lambrinou E., Lionis C., Abreu A., Savelieva I., Fumagalli S., Bo M., Rocca B. (2022). Frailty in cardiology: Definition, assessment and clinical implications for general cardiology. A consensus document of the Council for Cardiology Practice (CCP), Association for Acute Cardio Vascular Care (ACVC), Association of Cardiovascular Nursing and Allied Professions (ACNAP), European Association of Preventive Cardiology (EAPC), European Heart Rhythm Association (EHRA), Council on Valvular Heart Diseases (VHD), Council on Hypertension (CHT), Council of Cardio-Oncology (CCO), Working Group (WG) Aorta and Peripheral Vascular Diseases, WG e-Cardiology, WG Thrombosis, of the European Society of Cardiology, European Primary Care Cardiology Society (EPCCS). Eur. J. Prev. Cardiol..

[60] Fedeli U., Avossa F., Ferroni E., Saugo M., Pengo V. (2016). Contemporary Burden of Atrial Fibrillation and Associated Mortality in Northeastern Italy. Am. J. Cardiol..

[61] Lu L., Zhang Y., Chen C., Field R.W., Kahe K. (2022). Radon exposure and risk of cerebrovascular disease: A systematic review and meta-analysis in occupational and general population studies. Environ. Sci. Pollut. Res..

[62] Murphy A., Johnson C.O., Roth G.A., Forouzanfar M.H., Naghavi M., Ng M., Pogosova N., Vos T., Murray C.J.L., Moran A.E. (2018). Ischaemic heart disease in the former Soviet Union 1990–2015 according to the Global Burden of Disease 2015 Study. Heart.

[63] Bucholz E.M., Gooding H.C., de Ferranti S.D. (2018). Awareness of Cardiovascular Risk Factors in U.S. Young Adults Aged 18–39 Years. Am. J. Prev. Med..

[64] Ribeiro A.L.P., Duncan B.B., Brant L.C.C., Lotufo P.A., Mill J.G., Barreto S.M. (2016). Cardiovascular Health in Brazil: Trends and Perspectives. Circulation.

